# High-density visualization of antegrade fast pathway activation during atypical fast/slow atrioventricular nodal reentrant tachycardia in two cases

**DOI:** 10.1007/s10840-024-01858-z

**Published:** 2024-07-08

**Authors:** Shingo Maeda, Mihoko Kawabata, Tatsuaki Kamata, Yuhi Hasebe, Jackson J. Liang, Ruben Casado Arroyo, Kaoru Okishige, Hirotsugu Atarari, Koji Kumagai, Kenzo Hirao

**Affiliations:** 1 Department of Cardiovascular Medicine, Tohoku Medical and Pharmaceutical University, 1-15-1 Fukumuro Miyagino-Ku, Sendai, Miyagi 983-8536 Japan; 2 Arrhythmia Advanced Therapy Center, AOI Universal Hospital, Kawasaki, Japan; 3 Department of Cardiac Electrophysiology, University of Michigan, Ann Arbor, MI USA; 4 Department of Cardiology, Hôpital Erasme, Université Libre de Bruxelles, Brussels, Belgium; 5 Yokohama Minato Heart Clinic, Yokohama, Japan

Atrioventricular (AV) nodal reentrant tachycardia (AVNRT) is one of the most common regular tachycardia; however, approximately 6.4% of patients with AVNRT have atypical variants [1]. Though catheter ablation (CA) of a conventional slow pathway is an effective and safe treatment option for symptomatic patients with atypical AVNRT, retrograde atrial activation also becomes a target during CA. However, the components and mechanism of the reentrant circuit of atrioventricular (AV) nodal reentrant tachycardia (AVNRT) remain incompletely understood [2, 3]. Here, we present two cases in which high-density mapping enabled successful visualization of the fast pathway (FP) itself during atypical AVNRT, and these are the first reports delineating the antegrade FP conduction using 3D mapping.

A 62-year-old man (Case 1) and a 69-year-old man (Case 2) without structural heart disease were admitted for catheter ablation for symptomatic supraventricular tachycardia, which were diagnosed as atypical fast/slow AVNRTs. During the tachycardia, 3D electroanatomical mappings were performed using the Advisor™ HD Grid catheter and the EnSite cardiac mapping system (St Jude Medical, Inc., MN, USA) at the right atrial septum, and the antegrade FP conductions into the AV node at the top of the Triangle of Koch were reproducibly visualized in both cases (Fig. 1).

**Fig. 1:**
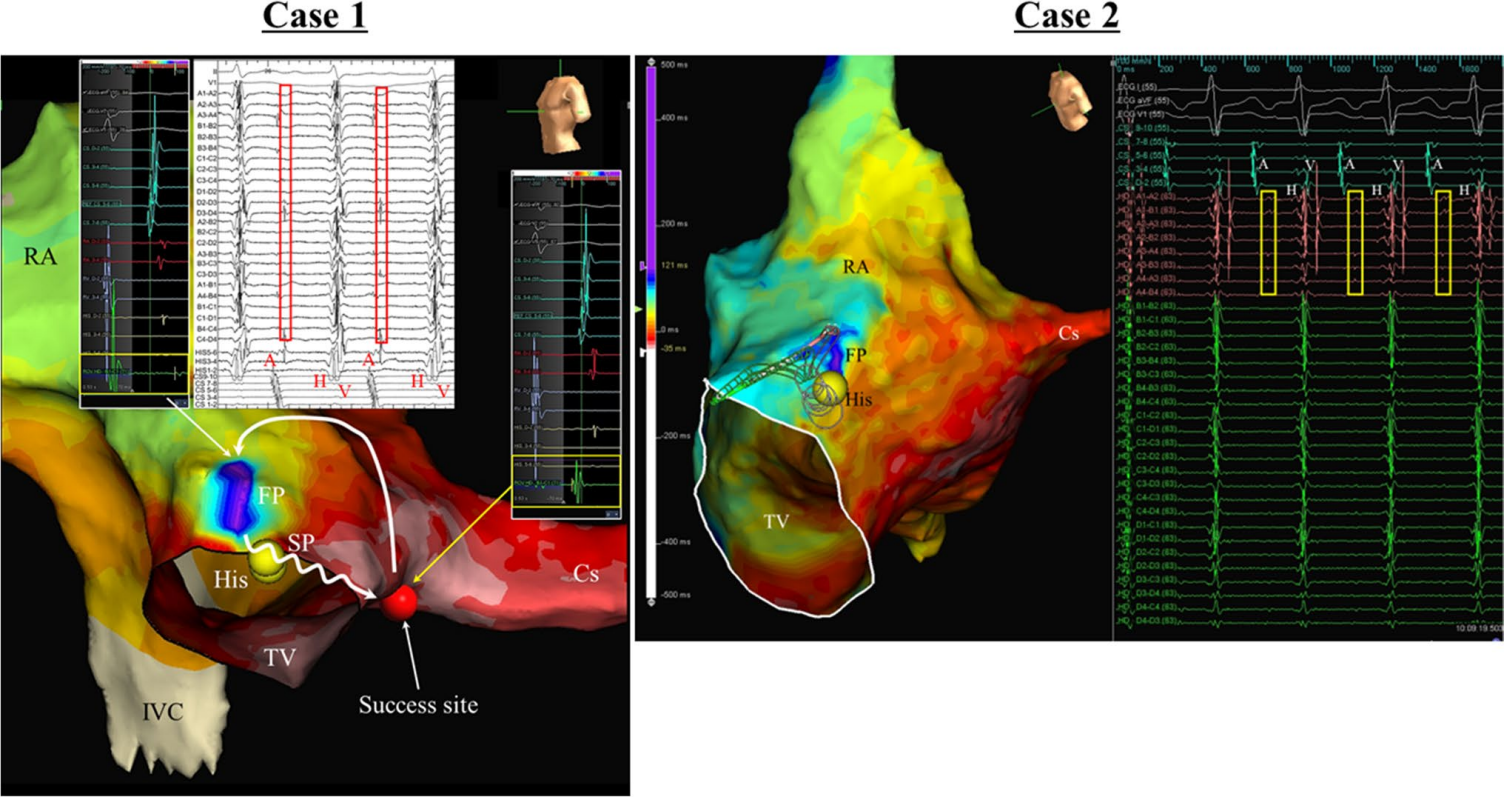
Activation mapping during fast/slow AVNRT. The red (Case 1) and yellow (Case 2) square shows the local electrograms at the FP and successful site. Cs, coronary sinus. FP, fast pathway; His, His bundle; IVC, inferior vena cava; RA, right atrium; SP, slow pathway; TV, tricuspid valve

These cases illustrate the ability of high-density mapping to allow a novel and clear visualization of the activation in the tachycardia circuit, especially the antegrade conduction through the FP. Several reports have been published regarding the utility of high-density mapping in patients with typical slow/fast AVNRT, in which the atrial activation was visualized showing the earliest atrial activation at the exit of the FP but no conduction of the retrograde conduction through the FP itself. Chua et al. reported the atrial activation in the Triangle of Koch with the HD mapping system in typical slow/fast AVNRT cases [4] in which the atrial propagation was successfully visualized after exiting the FP but not the FP activation. For typical slow/fast AVNRT, 3D mapping systems have been unable to acquire the tiny potentials reflecting the FP activation, which may be recorded very close to (or overlapping) the ventricular potential. In contrast, with atypical fast/slow AVNRT, 3D mapping systems may detect the antegrade conduction activity through the FP because the potential reflecting the FP activation is recorded after the timing of ventricular potential. Our cases are the first clinical report to describe the successful recording of the FP activation itself using high-density mapping. The use of multielectrode catheters and high-density mapping systems may be very helpful to clarify the enigmatic components and mechanisms of clinical AVNRT, especially atypical fast/slow AVNRT.

## Data Availability

The data that support the findings of this study are available from the corresponding author, upon reasonable request.
